# Tailored versus conventional surgical debridement in complex facial lacerations in emergency department: A retrospective study

**DOI:** 10.1097/MD.0000000000033572

**Published:** 2023-04-28

**Authors:** Byeong Kwon Park, Jin Hong Min, Jung Soo Park, Yeon Ho You, Won Joon Jeong, Yong Chul Cho, Se Kwang Oh, Yong Nam In, Hong Joon Ahn, Chang Shin Kang, Hyun woo Kyung, Joo Hak Kim, Ho Jik Yang, Byung Kook Lee, Heon Jong Yoo

**Affiliations:** a Department of Emergency Medicine, Chungnam National University Hospital, Jung-gu, Daejeon, Republic of Korea; b Department of Emergency Medicine, College of Medicine, Chungnam National University, Jung-gu, Daejeon, Republic of Korea; c Department of Emergency Medicine, Chungnam National University Sejong Hospital, Sejong, Republic of Korea; d Department of Plastic Surgery, Chungnam National University Sejong Hospital, Sejong, Republic of Korea; e Department of Plastic Surgery, College of Medicine, Chungnam National University, Jung-gu, Daejeon, Republic of Korea; f Department of Emergency Medicine, College of Medicine, Chonnam National University, Dong-gu, Gwangju, Republic of Korea; g Department of Emergency Medicine, Chonnam National University Hospital, Dong-gu, Gwangju, Republic of Korea; h Department of Obstetrics & Gynecology, Chungnam National University Sejong Hospital, Sejong, Republic of Korea; i Department of Obstetrics & Gynecology, College of Medicine, Chungnam National University, Jung-gu, Daejeon, Republic of Korea.

**Keywords:** complication, debridement, emergency department, facial laceration, local flap, scar, surgical debridement

## Abstract

Surgical debridement is an essential step in treating complex facial lacerations (CFL). As the CFL severity increases, conventional surgical debridement (CSD) of wound edges becomes difficult and may be insufficient. Because the severity and shape of each CFL vary, it is necessary to tailor the customized pre-excisional design, that is, tailored surgical debridement (TSD), for each case before performing surgical debridement. The use of TSD can enable effective debridement of CFL with higher severity. This study aimed to compare the cosmetic outcomes and complication incidence of CSD versus TSD according to CFL severity. In this retrospective observational study, eligible patients with CFL who visited the emergency department between August 2020 and December 2021 were examined. CFL severity was graded as Grades I and II. The outcomes of CSD and TSD were compared using the scar cosmesis assessment and rating (SCAR) scale, wherein a good cosmetic outcome was defined as a SCAR score of ≤ 2. The percentage of good cosmetic outcomes between the 2 groups was compared. The SCAR score and percentage of good cosmetic outcomes between the 2 groups were compared overall and by severity. For analyzing complication incidence, asymmetry, infection, and dehiscence incidence were compared. In total, 252 patients were enrolled [121 (48.0%) CSD and 131 (52.0%) TSD]. The median SCAR scores were 3 (1–5) and 1 (0–2) in all enrolled patients (*P* < .001), 2 (0–4), and 1 (0–1) in Grade I patients (*P* < .01), and 5 (4–6) and 1 (1–2) in Grade II patients (*P* < .001) in the CSD and TSD groups, respectively. The percentage of good cosmetic outcomes was 46.3% and 84.0% overall (*P* < .001), 59.6% and 85.0% in Grade I patients (*P* < .01), and 9.4% and 83.5% in Grade II patients (*P* < .001) in the CSD and TSD groups, respectively. The incidence of complications was significantly higher in the CSD group than in the TSD group, but this was limited to asymmetry. No significant difference was noted in infection or dehiscence. Compared with CSD, TSD can lead to an objectively good cosmetic prognosis at higher CFL severity and can reduce facial asymmetry occurrence.

## 1. Introduction

Facial lacerations (FL) with a variety of shapes and severities are reported among patients in the emergency department (ED).^[1]^ The principal goal of FL management is to close the wound to reduce healing time and the decrease the risk of further infection and scarring.^[2]^ However, scarring may occur even when an infection is prevented through wound closure. Since the face is well-exposed and conspicuous, reducing scarring is vital for an optimal cosmetic appearance and patient satisfaction.^[1,3]^ Scarring and infection can be more problematic with complex facial lacerations (CFL) than with simple, superficial FL. Studies on treatment methods for scar reduction in initial CFL cases are limited, and previous studies have not considered the CFL severity.^[4]^ Therefore, identification of an ideal closure method that considers CFL severity remains necessary.

In CFL treatment, debridement is more important than simple, superficial FL during the process leading to wound closure. Surgical debridement of the wound edges is an essential step in managing most CFLs. In preparing a CFL for suture, the wound edges that are appreciably damaged should be excised, converting a traumatic wound into a “clean” surgical wound.^[5,6]^ To achieve a more linear closure, removal of ragged wound edges or any sections of the wound that are de-vascularized requires a scalpel or sharp tissue scissors.^[5–7]^ If this debridement results in a slightly gaping wound, closure tension can be relieved by undermining the edges with sharp superficial dissection to the deep fascia.^[5]^ Conventional surgical debridement (CSD) proceeds as conservatively as possible without the need for customized designing for CFLs.^[5]^ Thus, tension and asymmetry can occur.^[5]^ Therefore, CSD may not effectively remove the entire ragged tissue, and transformation of the wound edge into a simplified overall linear shape may be difficult.^[5,7]^ As the severity of CFL increases, effective CSD becomes very difficult.^[5]^ If the tissue is preserved as much as possible, the possibility of preservation of damaged tissue also increases..^[5,7,8]^ However, if the debridement is excessive, it can leave gaping wounds, lead to tissue necrosis, or cause dehiscence because of excessive tension. These conditions become worse as the CFL severity increases.^[5,7,8]^

Given the different shape and size of the face and the different shape and severity of CFL across patients, customized pre-excisional designs (tailored surgical debridement [TSD]) should be tailored according to each CFL case before performing surgical debridement. ^[5,9–14]^ In TSD, the area to be excised is delineated using a skin marker pen before excisional debridement is performed, to obtain the best results. TSD can effectively remove almost all damaged tissues and create clean and simplified wound edges. ^[5,12–14]^ However, while applying TSD, excessive tension or facial asymmetry may occur, which can be resolved by applying a customized pre-excisional design, such as a local flap design (LFD).^[5,12–14]^ Therefore, the overall prognosis may be more favorable, which may result in a favorable prognosis even if the severity of CFL increases.^[5,13,14]^

Although the design method of debridement has been studied, only few studies have compared TSD and CSD, especially according to CFL severity.^[10,15,16]^ Therefore, this study aimed to compare the cosmetic outcomes and complication incidence of CFLs between TSD and CSD, according to CFL severity.

## 2. Methods

### 2.1. Study design and patients

In this retrospective observational study, we used wound registry data collected from patients with FL who visited the ED of Chungnam National University Sejong Hospital, a university-affiliated 409-bed care referral center in Sejong, South Korea, and who underwent wound closure between August 2020 and December 2021. The Institutional Review Board of Chungnam National University Hospital approved this study (approval number: CNUSH IRB 2022-02-005), and written informed consent was obtained when registering for wound registry from all patients in accordance with national requirements and the principles of the Declaration of Helsinki and registered in a database.

Patients who visited the ED with FL were included. The exclusion criteria were as follows: patients who were aged < 18 years, opposed wound registry registration, took medication for chronic skin disease, had open fractures at the laceration site, and had degloving injuries. Patients with FL with superficial or sharp wound edges were also excluded.

CFLs were classified into Grades I and II according to severity, conditions of wound edges, and laceration shapes (Table 1).

**Table 1:** Grade of complex facial lacerations according to severity.

Classification	Description
Grade I	Moderate ragged macerated, and linear
	Macerated or ragged wound edges <2 mm from the lacerated line
	Beveled cross section in the lacerated edge
	Mild tissue loss below the epidermal layer in the lacerated edge
Grade II	Severe ragged, macerated, or nonlinear*
	Macerated or ragged wound edges >2 mm from the lacerated line
	Moderate to severe tissue loss below the epidermal layer from the lacerated edge
	Partially avulsed segment in wound edge from the lacerated edge

* Lacerations consisting multiple lines defined nonlinear.

### 2.2. Interventions

CSD is aimed at approximation by conservative sharp debridement. Therefore, CSD was proceeded without drawing an excisional line, and debridement was performed only to the extent that the approximation was possible.^[5,14]^ Meanwhile, TSD is aimed at approximate tissues with minimal injuries by removal beyond the severely macerated, ragged wound edge or partially avulsed segment in the wound edge.^[13,14]^ After the bleeding was controlled, a skin marker pen (Dual Marking Pen, Ayida, Xiamen, Fujian, China) was used to draw the design according to the aforementioned goal, and wound excision and incision were performed.^[13,14]^ Various types of LFD were applied in cases of excessive tension or when preserving facial anatomical symmetry or function was required.^[13,14]^ For all procedures performed in the ED, 6 to 0 Mersilk (Ethicon, Somerville, NJ) was used to close the cutaneous layer, 6 to 0 Monosyn (B. Braun, Rubi, Barcelona, Spain) was used for the subcutaneous layer, and 5 to 0 coated VICRYL (Ethicon, Somerville, New Jersey) was used for closure below the subcutaneous layer.

### 2.3. Outcomes evaluation

The primary outcome of this study was comparison of the long-term cosmetic outcomes of TSD versus CSD. The primary outcome was compared using the scar cosmesis assessment and rating (SCAR) score between the TSD and CSD groups (Table S1, Supplemental Digital Content, http://links.lww.com/MD/I825).^[17]^ In the plastic surgery outpatient clinic, SCAR scores were recorded between 6 months and 1 year after repair, and these scores were recorded on the outpatient chart and in the wound registry with photographs. The percentages of good prognoses between the 2 groups were also compared as a primary outcome. A good cosmetic outcome was defined as a SCAR score of ≤ 2.

The secondary outcome was comparison of the incidence of complications such as asymmetry, infection, and dehiscence between the 2 groups.

### 2.4. Analysis

Statistical analyses were performed using SPSS version 21.0 (IBM Corp., Armonk, NY) to compare the TSD and CSD groups. Nominal variables are expressed as frequencies (percentages), and Fisher exact test was used for the analysis. Continuous variables were tested for normal distributions using the Shapiro–Wilk test. Non-normally distributed variables are expressed as median values (interquartile ranges), whereas normally distributed variables are described as means (± standard deviations). Student *t* test was used for normally distributed data, whereas the nonparametric Mann–Whitney *U* test was used for non-normally distributed data. Statistical significance was set at *P* < .05.

## 3. Results

### 3.1. Characteristics of the enrolled patients

**Figure 1.**
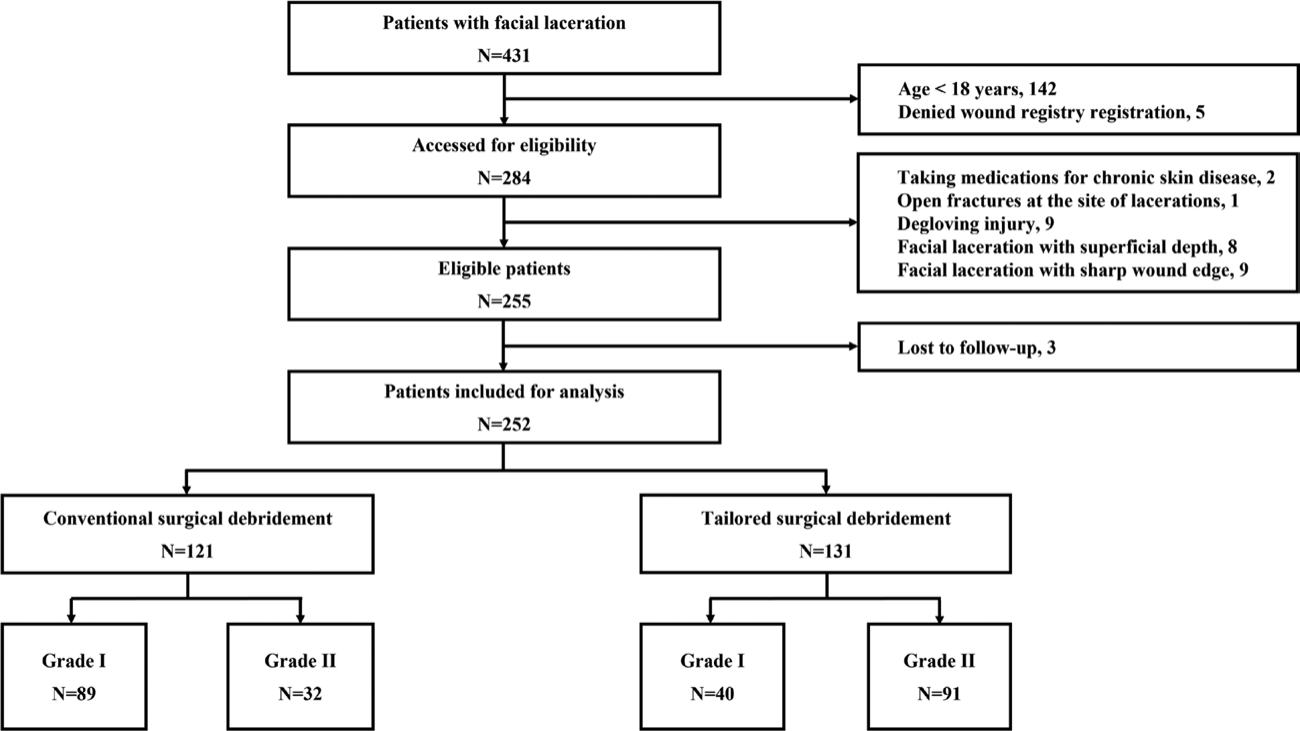
Flow diagram of patient selection in this study.

In total, 431 patients visited the ED for FL repair. Following exclusions, 284 patients were included in the study. Then, 29 patients were further excluded based on the exclusion criteria. Three of the remaining 255 patients were lost to follow-up. Eventually, 252 patients were enrolled and analyzed, among whom 121 (48.0%) underwent CSD and 131 (52.0%) underwent TSD (Fig. 1).

No significant differences were noted in age, sex, incidence of hypertension and diabetes mellitus, smoking, and alcohol intake between the CSD and TSD groups (Table 2). In addition, no significant differences were observed in injury to repair time, laceration length and depth, angle of laceration to the relaxed skin tension line, laceration region, and laser scar therapy between the CSD and TSD groups (Table 2). Although a significant difference was noted in procedure duration, no significant difference was noted between the CSD and TSD groups when divided by grade (Table 2).

**Table 2:** General characteristics of the patients who visited the emergency department with complex facial laceration.

Characteristic	Cohort (n = 252)	CSD (n = 121)	TSD (n = 131)	*P* value
Age (y), median (IQR)	48.0 (34.0–60.0)	48.0 (35.0–61.0)	50.0 (34.0–59.0)	.811
Male sex, n (%)	172 (68.3)	82 (67.8)	90 (68.7)	.874
Hypertension, n (%)	33 (13.1)	18 (14.9)	15 (11.5)	.422
Diabetes mellitus, n (%)	17 (6.7)	9 (7.4)	8 (6.1)	.674
Smoking, n (%)	33 (13.1)	14 (11.6)	19 (14.5)	.507
Alcohol, n (%)	105 (41.7)	52 (43.0)	53 (40.5)	.645
Injury to repair time (h), median (IQR)	2.0 (1.0–4.9)	2.0 (1.0–4.0)	2.0 (1.0–6.8)	.985
FL Length (cm), median (IQR)	3.0 (2.0–4.0)	2.5 (1.5–4.0)	3.0 (2.0–4.0)	.053
Depth of FL				.120
Subcutaneous	170 (67.5)	87 (71.9)	83 (63.4)	
Muscle	54 (21.4)	24 (19.8)	30 (22.9)	
Periosteum	28 (11.1)	10 (8.3)	18 (13.7)	
Angle to the RSTL (°), median (IQR)	40 (0–70)	45 (0–70)	30 (0–60)	.662
FL region, n (%)				.475
Forehead	89 (35.3)	42 (34.7)	47 (35.9)	
Periorbital area	47 (18.7)	30 (24.8)	17 (13.0)	
Nose	33 (13.1)	13 (10.7)	20 (15.3)	
Cheeks	20 (7.9)	10 (8.3)	10 (7.6)	
Perioral area	34 (13.5)	11 (9.1)	23 (17.6)	
Chin	29 (11.5)	15 (12.4)	14 (10.7)	
Procedure duration, median (IQR)	40.0 (30.0–60.0)	40.0 (30.0–50.0)	50.0 (40.0–60.0)	.001
Grade I, Median (IQR)	30.0 (30.0–46.5)	30.0 (30.0–42.8)	40.0 (30.0–50.0)	.229
Grade II, Median (IQR)	50.0 (40.0–60.0)	55.0 (40.0–60.0)	50.0 (40.0–60.0)	.575
Laser scar therapy, n (%)	65 (25.8)	30 (24.8)	35 (26.7)	.728
Complications, n (%)	6 (2.4)	6 (5.0)	0 (0.0)	.010
Asymmetry, n (%)	4 (1.6)	4 (3.3)	0 (0.0)	.036
Infection, n (%)	2 (0.8)	2 (1.7)	0 (0.0)	.140
Dehiscence, n (%)	2 (0.8)	2 (1.7)	0 (0.0)	.140

CSD = conventional surgical debridement, FL = facial laceration, IQR = interquartile range, RSTL = relaxed skin tension line, TSD = tailored surgical debridement.

### 3.2. Main results

**Figure 2.**
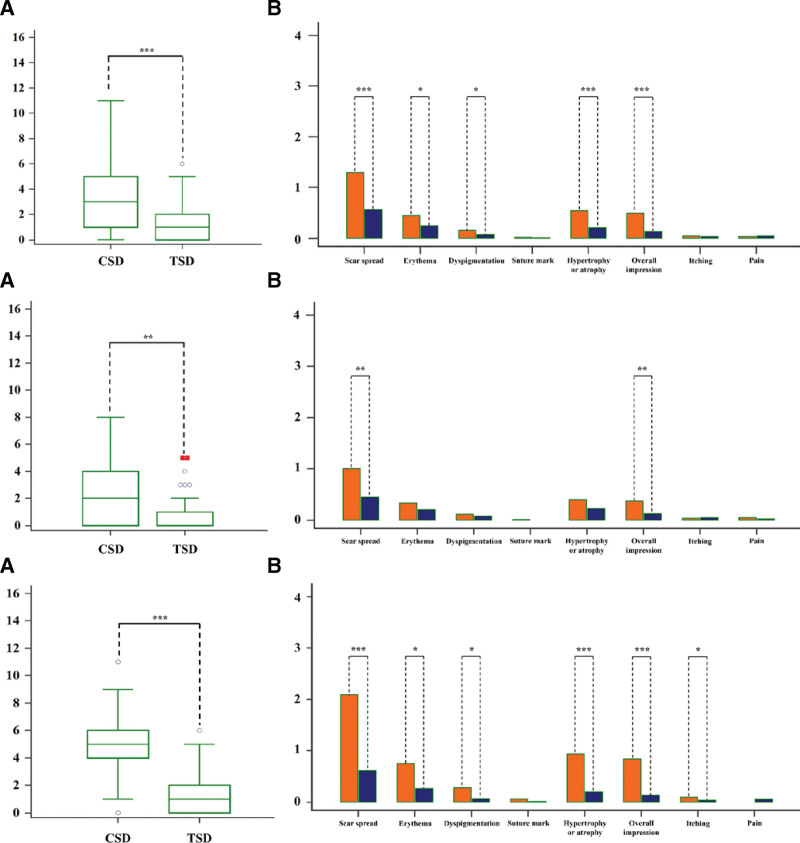
Comparisons of the total SCAR score and parameters between the CSD and TSD groups. (A) All enrolled patients: (A) comparisons of the total SCAR score, (B) comparisons of SCAR parameters. Orange, CSD; Navy, TSD (B) Grade I patients: (A) comparisons of the total SCAR score, (B) Comparisons of SCAR parameters. Orange, CSD; Navy, TSD (C) Grade II patients: (A) comparisons of the total SCAR score, (B) comparisons of SCAR parameters. Orange, CSD; Navy, TSD. (* *P *< .05, ** *P *< .01, *** *P *< .001). CSD = conventional surgical debridement, SCAR = scar cosmesis assessment and rating, TSD = tailored surgical debridement.

The median SCAR scores were 3 (1–5) in the CSD group and 1 (0–2) in the TSD group (*P* < .001; Fig. 2). For Grade I patients, the median SCAR scores were 2 (0–4) in the CSD group and 1 (0–1) in the TSD group (*P* < .01; Fig. 2). For Grade II patients, the median SCAR scores were 5 (4–6) in the CSD group and 1 (1–2) in the TSD group (*P* < .001; Fig. 2).

Regarding parameters on the SCAR scale, scar spread, erythema, dyspigmentation, hypertrophy or atrophy, and overall impression were significantly lower in the TSD group than in the CSD group (Fig. 2). Scar spread and overall impression were also significantly lower in the TSD group than in the CSD group for Grade I patients (Fig. 2). Scar spread, erythema, dyspigmentation, hypertrophy or atrophy, overall impression, and itching were significantly lower in the TSD group than in the CSD group for Grade II patients (Fig. 2).

**Figure 3.**
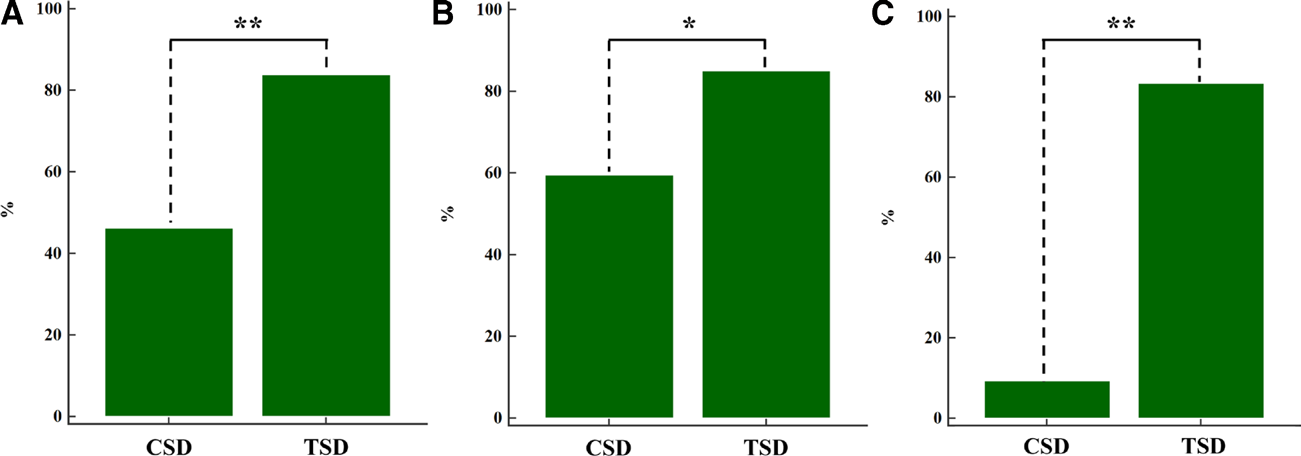
Percentage of good cosmetic outcomes in the CSD and TSD groups. (A) All enrolled patients. (B) Grade I patients. (C) Grade II patients. (* *P *< .01, ** *P *< .001). CSD = conventional surgical debridement, TSD = tailored surgical debridement.

A good cosmetic outcome was achieved in 46.3% of patients in the CSD group and 84.0% of patients in the TSD group (*P* < .001; Fig. 3). In Grade I patients, an excellent cosmetic outcome was achieved in 59.6% of patients in the CSD group and 85.0% of patients in the TSD group (*P* < .01; Fig. 3). In Grade II patients, a good cosmetic outcome was achieved in 9.4% of patients in the CSD group and 83.5% of patients in the TSD group (*P* < .001; Fig. 3).

The incidence of complications was significantly lower in the TSD group than in the CSD group (*P* = .010; Table 2). Although a significant difference was noted in the incidence of complications, it was only in terms of asymmetry, no significant difference was noted in the incidence of in infection or dehiscence (Table 2). Asymmetry occurred only in the CSD group for Grade II patients. Infection occurred in 1 patient each among Grade I and II patients in the CSD group. Dehiscence occurred in the same patient as the infection.

## 4. Discussion

This study compared the cosmetic outcomes and complication incidence of CSD versus TSD according to CFL severity, revealing that when severity increased, TSD provided better cosmetic outcomes and reduced complications such as asymmetry (Figs. 2 and 3, Table 2).

**Figure 4.**
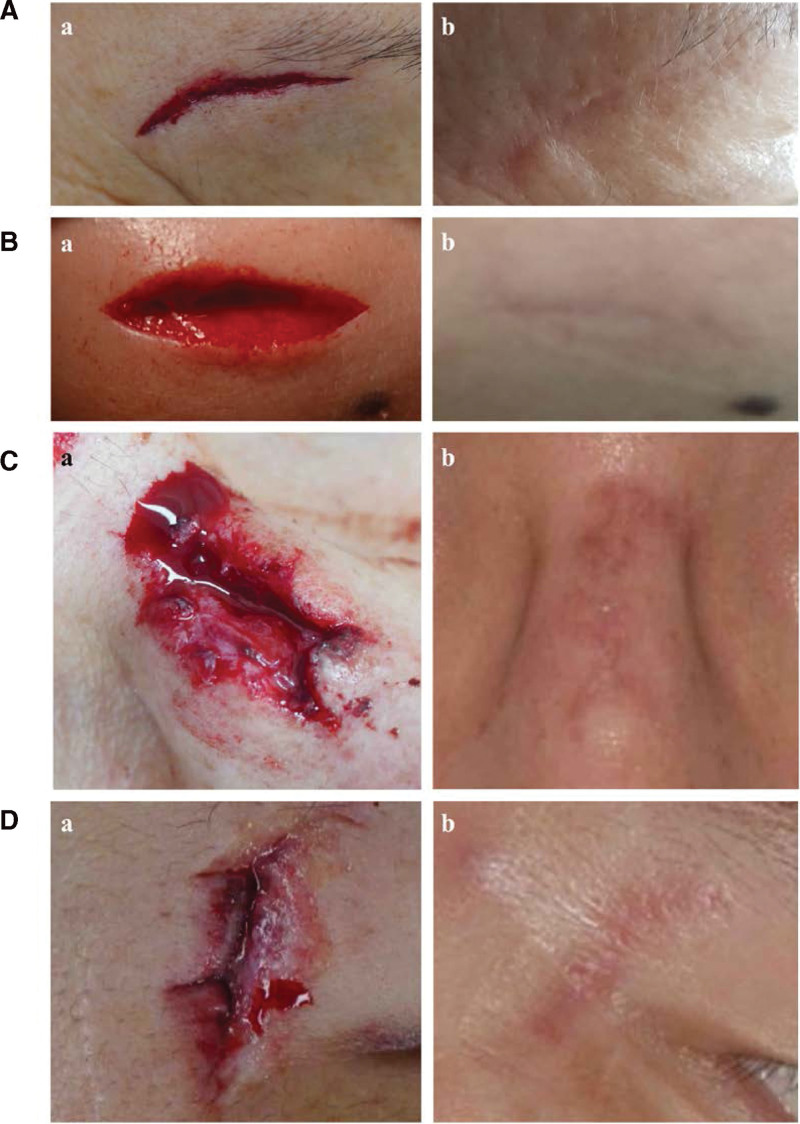
Outcomes of CSD for CFL. (A) Findings of a 57-year-old woman who visited the ED for a periocular area laceration (Grade I): (A) the CFL has beveled wound edges, and partial tissue loss is noted below the epidermal layer in the lacerated edge, (B) the clearly visible atrophic scar 6 months after stitch removal. (B) Findings of a 29-year-old woman who visited the ED for a chin laceration (Grade I): (A) the CFL has beveled wound edges, and mild tissue loss is noted below the epidermal layer in the lacerated edge, (B) the visible atrophic scar 8 months after stitch removal. (C) Findings of a 39-year-old man who visited the ED for a nasal area laceration (Grade II): (A) The CFL has nonlinear ragged wound edges > 2 mm from the lacerated line, (B) seven months after stitch removal with an undesirable scar, including severe spread, dyspigmentation, and severe atrophy. (D) Findings of a 39-year-old man who visited the ED for a periocular area laceration (Grade II): (A) wound condition at the time of admission. The CFL has nonlinear ragged wound edges > 2 mm from the lacerated line, (B) eight months after stitch removal with an undesirable scar, including severe spread, deep red erythema, and moderate atrophy. CFL = complex facial laceration, CSD = conventional surgical debridement, ED = emergency department.

**Figure 5.**
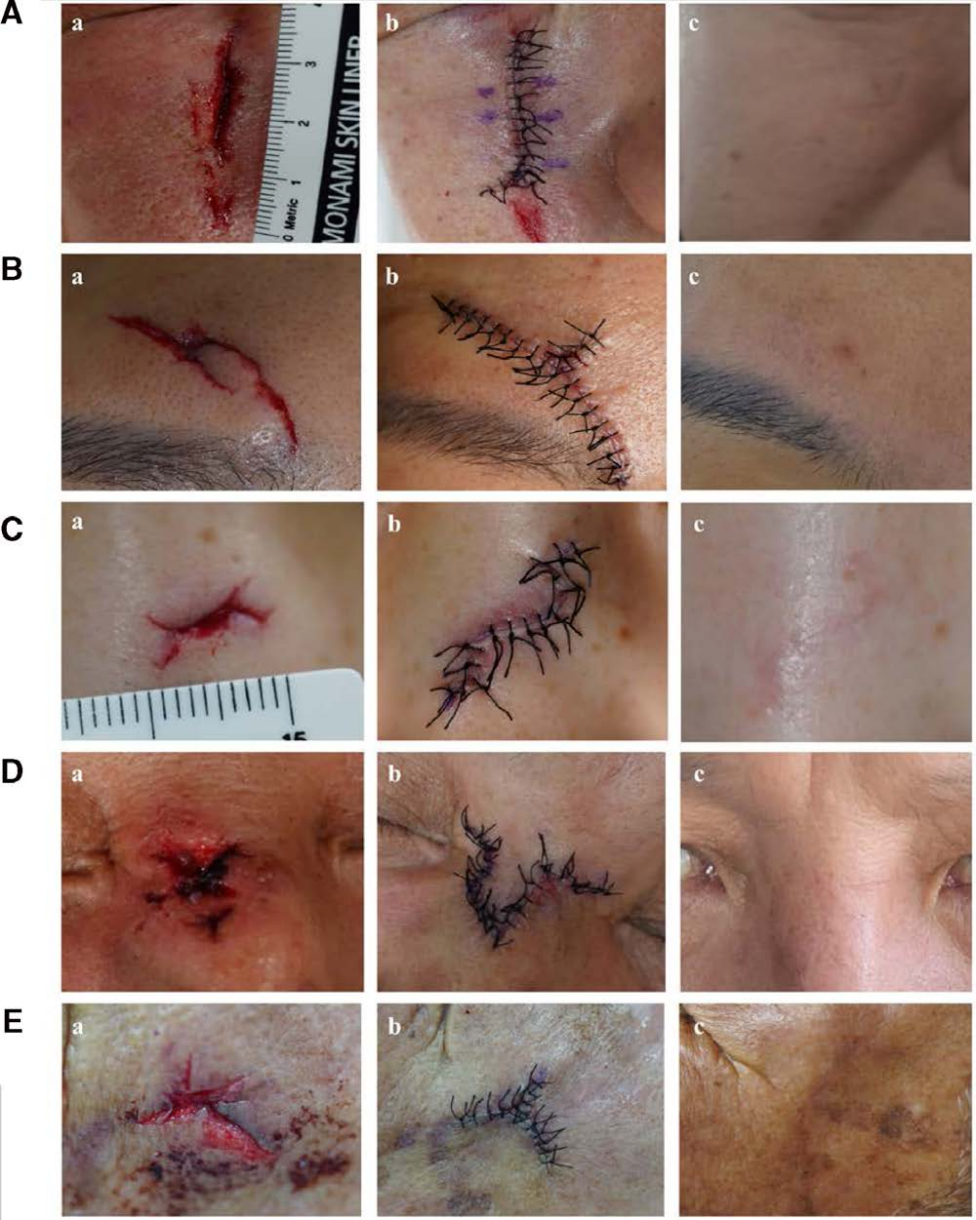
Outcomes of TSD for CFL. (A) Findings of a 60-year-old man who visited the ED for a cheek laceration (Grade I): (A) the CFL has macerated and ragged wound edges < 2 mm from the lacerated line, (B) condition after suturing. The L-shape excisional design is applied for removing the ragged wound edge and ensuring facial symmetry, and (C) six months after stitch removal, the scar is no longer visible. (B) Findings of a 38-year-old woman who visited the ED for a forehead laceration (Grade II): (A) the CFL has a nonlinear shape with an avulsed flap, (B) condition after suturing. A–T excisional design is applied to remove the avulsed flap. (c) Nine months after stitch removal where the scar is no longer visible. (C) Findings of a 38-year-old woman who visited the ED for a nasal bone area laceration (Grade II): (a) The CFL has nonlinear and avulsed flaps at both ends of the wound with severe subcutaneous tissue loss. (b) Condition after suturing. Hexagonal incision and transposition are performed to remove the avulsed flap and to cover subcutaneous tissue loss. (c) Six months after stitch removal, (light pink) erythema is noted. (D) Findings of a 55-year-old man who visited the ED for a nasal area laceration (Grade II). (a) The CFL has a nonlinear ragged wound edge, and severe tissue loss is noted below the epidermal layer from the lacerated edge. (b) Condition after suturing. A rhomboid transposition flap is used to remove the ragged lacerated edge, simplifying complex shapes and covering subcutaneous tissue loss. (c) Nine months after stitch removal where the scar is no longer visible. (E) Findings of a 78-year-old man who visited the ED for a periocular area laceration (Grade II): (a) The CFL has a nonlinear avulsed segment at the wound edge, (B) condition after suturing. A-T excision is performed to remove the avulsed segments and simplify complex shapes, and (C) ten months after stitch removal where the scar is no longer visible. CFL = complex facial laceration, ED = emergency department, TSD = tailored surgical debridement.

CFLs are nonlinear, consisting of multiple lines, sometimes satellite macerated or ragged wound edges. In addition, healing is deteriorated by devitalized and contaminated tissues (Figs. 4 and 5). Therefore, CFL can lead to ugly scars and thus have an impact on psychosocial functioning, causing increased anxiety and self-consciousness and impairing social functioning and emotional well-being (Figs. 4 and 5).^[18,19]^

Laceration healing comprises the following 3 major phases: inflammation, proliferation, and remodeling.^[20]^ In the inflammatory phase, the more severe the debris, the more devitalized, nonviable, and contaminated tissues noted in the wound and the worse the inflammatory response. Increased inflammation causes over-proliferation and over-differentiation of cells (such as fibroblasts and keratinocytes) in the proliferation phase at the wound site. Additionally, collagen production is increased by excessive fibroblasts. In the remodeling phase, dysregulated inflammatory mediators can cause excessive extracellular matrix synthesis by disorganized collagen bundles. Collectively, these result in excessive, obtrusive, and undesirable scarring. ^[20,21]^ Minimizing the inflammatory response during the wound healing process is the simplest way to reduce scarring, and this can be achieved by effective debridement. Through debridement, devitalized and nonviable tissue, gross contaminants, and foreign bodies are removed, creating a wound edge as close as possible in cleanliness to healthy tissue.^[20,21]^

Even if the inflammatory reaction is reduced by effective debridement, this alone is not enough. Even if the epidermis looks relatively clean, if the dermal layer of the lacerated edge is a beveled cross section or has dermal injuries, depressed or indented scars can occur even if the wound edges are relatively clean, and the degree of raggedness is less severe (Fig. 4).^[5,6,8,19,22]^ This becomes more prominent in case of damage to the subcutaneous tissue.^[8,22]^ Therefore, these damages must be corrected to reduce scarring. To this end, the beveled cross section should be made perpendicular through sharp excisional debridement, and if there is damage to the subcutaneous tissue, it should also be repaired.^[5,6,8,14,23]^ In correcting damage through debridement, excessive tension, gaping, and facial asymmetry may occur.^[5,12–14]^

In treating the CFL, sufficient debridement should be performed to reduce scarring. Most surgeons and physicians mainly use CSD for CFL repair. However, CSD may not be sufficient to remove the entire ragged tissue effectively, and it may be difficult to turn the wound edge into a simplified overall linear shape.^[5–7]^ If the shape of the CFL is complicated, debridement is limited, and wound closure is performed as conservatively as possible.^[5,7,8]^ If the tissue is preserved as much as possible, even the severely damaged tissue can be preserved.^[5,8,20,21,23]^ For this reason, when CSD is performed alone for CFLs with nonlinear or harsh ragged edges, it is highly probable that devitalized, contaminated, and badly damaged tissues are retained.^[5,13,19,21,23]^ This may increase the inflammatory response, leading to excessive wound healing, increased dermal fibrosis, disorganized collagen, disappearance of elastic fibers and appendages, and disruption of skin texture, thereby creating unsightly scars.^[20,21]^ Macerated or ragged wound edges are excised; usually, 1 to 2 mm is sufficient.^[5,8]^ However, it can be widened depending on its severity. If the debridement is too much, it can leave gaping wounds, cause tissue necrosis, or lead to dehiscence due to excessive tension. ^[5,8,11–15]^ These are thought to become more severe as the severity of CFL increases. Therefore, debridement for CFL needs planning before excision in terms of using TSD based on individual CFL cases, with the goal of safer surgeries and more favorable outcomes. ^[5,12–15]^ This becomes more important as the severity increases.

Given that each patient has a different face shape and different CFL severity, it is important to tailor the pre-excisional design for debridement according to each CFL case before performing surgical debridement.^[5,10–15]^ In TSD, the area to be excised is custom-made and designed before debridement is performed.^[13,14]^ Before drawing a design on the skin, surgeons should plan ahead and draw a design that can produce the best outcomes by considering the possible complications such as asymmetry, gaping, and excessive tension.^[13,14]^ LFD may be applied in some cases to enable laceration closure with significantly reduced tension and reduced gaping.^[13,14]^ By doing so, TSD can effectively remove almost all damaged tissues and create clean and simplified wound edges and a smooth shape (Fig. 5). Therefore, the overall prognosis may be more favorable even if the CFL severity increases.^[13,14]^

In this study, we evaluated scars using the SCAR scores, as this scale was created to evaluate postsurgical scars.^[17]^ Several scar scales, such as the Vancouver scar scale, the patient and observer scar assessment scale, the Manchester scar scale, and the Stony Brook scar evaluation scale, have been used to evaluate the condition of scars. Each scale has its advantages and disadvantages in assessing the different characteristics of scars. However, no valid and reliable scar scale is currently available to effectively assess postsurgical scar quality. The Vancouver scar scale and patient and observer scar assessment scale were originally developed to assess burn scars and are unsuitable for assessing postsurgical scars.^[24]^ The Stony Brook scar evaluation scale lacks a subjective parameter, thus limiting its clinical utility.^[25]^ The Manchester scar scale has been criticized for being better suited to assess linear scars and not account for symptoms.^[25]^ Therefore, an evaluation tool that provides a reliable outcome measure for postsurgical scars is needed. The SCAR scale can be used to assess postsurgical scars in a clinical and research context. The convergent validity, inter-rater reliability, and intra-rater reliability of the SCAR scale have been tested, and the results showed that this scale is outstanding in terms of feasibility, validity, and reliability for postoperative scar-related outcome measurements. ^[17,24]^ After a short training period, the SCAR scale can be quickly and reliably used during the clinical follow-up process.^[17,24]^

In our analysis, when the cosmetic prognoses of CSD and TSD were compared using the SCAR scale, the TSD group showed a significantly better prognosis across the entire cohort than the CSD group. Even for grades classified according to the CFL severity, the CSD group had a better prognosis than the TSD group. The prognostic difference between the CSD and TSD groups in Grade II patients with higher severity was significantly larger. Moreover, a marked difference was noted in the proportion of patients with good cosmetic outcomes among Grade II patients. The proportion of patients with good cosmetic outcomes was higher in the TSD group than in the CSD group. This result was more marked in Grade II patients. These results indicate that TSD can produce cleaner and sharper edges with reduced skin tension than CSD.

We found significant differences in the SCAR scale parameters between both the groups (Fig. 4). Among the parameters for Grade I patients, scar spread and overall impression were significantly different between CSD and TSD. For Grade II patients, additionally significant differences were noted in erythema, dyspigmentation, hypertrophy or atrophy, and itching (Fig. 4). Extended scar spread is the result of a rupture of the dermis and excessive tension. Erythema results from increased local blood flow and vascular permeability of capillaries stimulated by inflammatory cytokines.^[26,27]^ Dyspigmentation may result from inflammatory conditions, and hypertrophic scars result from the excessive proliferation of myofibroblasts and increased collagen deposition within the scar.^[26,28]^ As an adjunct to collagen production, the synthesis of histamine is increased, and the response of histamine receptors is activated, resulting in pruritus.^[27]^ Furthermore, various substances such as acetylcholine, bradykinin, and proteinases are involved in pruritic sensations.^[29]^ This also means that, compared to CSD, TSD can lower the inflammatory response in the wound healing process and approximate the wound edge by making it a relatively intact edge. This suggests that debridement is required for CFLs with damaged tissue and that TSD is more effective than CSD as the CFL severity increases.

Regarding complications, asymmetry showed a significant difference between the 2 groups. Asymmetry occurred only in Grade II patients in the CSD group. Asymmetry is caused by excessive tension that leads to asymmetry during approximation and scar contracture during wound healing. ^[5,13,14,19]^ If asymmetry is likely to occur, a design that can correct this should been applied, such as applying LFD in the TSD group in some cases. It seems that the CSD group lacks such processes. In terms of infection, no significant difference was noted between the CSD and TSD groups. Infection and dehiscence occurred in the same patient. This is thought to occur because both the procedures effectively prevent infection through debridement.

This study has some limitations. First, it was retrospective in nature and was conducted at a single center. A prospective multicenter and multiethnic study with a larger sample size is needed for generalization of our study findings. Second, self-fulfilling prophecy bias was possible, as treating physicians or surgeons were exposed to the results of TSD and CSD.

In conclusion, for CFL with higher severity, when TSD is properly applied considering the anatomical symmetry and function of the face, objectively good cosmetic outcomes and subjective patient satisfaction can occur. However, although there was no difference in infection and dehiscence when the severity of CFL is high, CSD is more likely to lead to asymmetry than TSD.

## Author contributions

**Conceptualization:** Byeong Kwon Park, Jin Hong Min.

**Data curation:** Byeong Kwon Park, Jung Soo Park.

**Formal analysis:** Yeon Ho You.

**Funding acquisition:** Won Joon Jeong, Yong Chul Cho.

**Investigation:** Se Kwang Oh, Yong Nam In.

**Methodology:** Jin Hong Min.

**Project administration:** Hong Joon Ahn, Chang Shin Kang.

**Resources:** Byung Kook Lee.

**Supervision:** Joo Hak Kim, Ho Jik Yang.

**Software:** Heon Jong Yoo.

**Validation:** Hyun Woo Kyung, Joo Hak Kim, Ho Jik Yang.

**Writing – original draft:** Jin Hong Min.

**Writing – review & editing:** Byeong Kwon Park, Jin Hong Min.

## Supplementary Material


