# Supplementary material for: Tailored versus conventional surgical debridement in complex facial lacerations in emergency department: A retrospective study
Source: Medicine (Baltimore). 2023 Apr 28;102(17):e33572. doi: 10.1097/MD.0000000000033572 (PMC10145807; doi:10.1097/MD.0000000000033572)
Supplement: Supplementary file 1 [file medi-102-e33572-s001.pdf]

Tailored versus conventional surgical debridement in complex facial lacerations in emergency department: a retrospective study

Byeong Kwon Park et al.

Supplemental Table 1. The Scar Cosmesis Assessment and Rating Scale

Parameter	Descriptor	Score
**Clinician questions**		
Scar spread	None/near invisible	0
	Pencil-thin line	1
	Mild spread, noticeable on close inspection	2
	Moderate spread, obvious scarring	3
	Severe spread	4
Erythema	None	0
	Light pink, some telangiectasias may be present	1
	Red, many telangiectasias may be present	2
	Deep red or purple	3
Dyspigmentation	Absent	0
	Present	1
Suture marks	Absent	0
	Present	1
Hypertrophy/atrophy	None	0
	Mild: palpable, barely visible hypertrophy or atrophy	1
	Moderate: clearly visible hypertrophy or atrophy	2
	Severe: marked hypertrophy or atrophy or keloid formation	3
Overall impression	Desirable scar	0
	Undesirable scar	1
**Patient's questions**		
Itch	No	0
	Yes	1
Pain	No	0
	Yes	1

The scale measures the scar cosmesis, and the scores range from 0 (best possible scar) to 15 (worst possible scar).
